# An unfolded protein response-inducible protein (ULI1) mediates aromatic alcohols biosynthesis through reinforcing the Ehrlich pathway in *Saccharomyces cerevisiae*

**DOI:** 10.3389/fmicb.2026.1934502

**Published:** 2026-09-14

**Authors:** Xiaowei Gong, Yong Shen, Shanzhai Shang, Jianbo Zhan, Guanghui Ma, Huajun Luo, Shiwei Li, Chunxia Song, Donglai Zhu, Yiyong Luo

**Affiliations:** 1 R&D Center of China Tobacco Yunnan Industrial Co., Ltd., Kunming, China; 2 National R&D Center for Freshwater Fish Processing, College of Life Sciences, Jiangxi Normal University, Nanchang, China; 3 Yunnan Forestry Technological College, Kunming, China; 4 Faculty of Life Science and Technology, Kunming University of Science and Technology, Kunming, China

**Keywords:** 2-phenylethanol, gene editing, proteomics, *Saccharomyces cerevisiae*, transcriptomics, tryptophol, unfolded protein response

## Abstract

Aromatic alcohols such as 2-phenylethanol (2-PE) and tryptophol (TOL) possess diverse biological functions. Here, an unfolded protein response-inducible gene *ULI1* and an aldehyde dehydrogenase gene *ALD3* in *Saccharomyces cerevisiae* were disrupted using the CRISPR-Cas9 system. Fermentation assays revealed that both the *ULI1* frameshift (Kmuli1) and knockout (KΔuli1) mutants exhibited significantly reduced 2-PE and TOL yields relative to the wild-type strain KMLY1-6, retaining 46.32 and 64.54% for 2-PE, and 34.61 and 77.73% for TOL, respectively. In contrast, *ALD3* knockout moderately elevated 2-PE and TOL production by 4.98 and 20.57%, respectively. These results indicate that both *ULI1* and *ALD3* mediate the biosynthesis of 2-PE and TOL in *S. cerevisiae*. To further confirm the role of *ULI1*, three chromosomal integration expression (CIE) strains (KXuli1, KXIIuli1, KΔald3uli1) and one plasmid-based expression (PBE) strain (BY4741/pY26-uli1) were constructed. Compared with the control KMLY1-6, the CIE strains increased 2-PE and TOL production by 10.13–12.91% and 25.76–29.28%, respectively. Meanwhile, the PBE strain produced significantly higher levels of 2-PE and TOL than the empty vector control (BY4741/pY26), with maximum increases of 38.80% and 30.86% detected at 60 h of cultivation. To further elucidate the molecular mechanism of *ULI1* in 2-PE and TOL biosynthesis, comparative transcriptomic analysis for Kmuli1 *vs.* KMLY1-6, together with transcriptomic and proteomic analyses for BY4741/pY26-uli1 *vs.* BY4741/pY26, was performed. Multi-omics results revealed that ULI1 reinforces the Ehrlich pathway, modulates metabolic flux, maintains mitochondrial structure and cellular protein homeostasis, and coordinates stress responses with metabolic redistribution. Collectively, ULI1 acts as a novel regulatory hub linking stress tolerance and cellular homeostasis, and facilitates aromatic alcohol biosynthesis by strengthening the Ehrlich pathway in *S. cerevisiae*.

## 1 Introduction

Aromatic alcohols, a distinct class of aromatic compounds, are predominantly biosynthesized in natural systems via the metabolic transformation of aromatic amino acids. These compounds possess great economic value and research potential due to their versatile biological functions. For instance, 2-phenylethanol (2-PE), a metabolite with a pleasant rose-like fragrance, is widely used in the food, cosmetics, and tobacco industries (Hua and Xu, 2011; Bernardino et al., 2024). Additionally, it acts as a quorum-sensing molecule in yeast (Chen and Fink, 2006) and exhibits broad-spectrum antibacterial and antifungal activities (Fraud et al., 2003; Mo and Sung, 2007; Wang et al., 2020), thus garnering considerable research attention. Tryptophol (TOL), another representative aromatic alcohol, also boasts multiple biological functions, including the inhibition of cytokine production, the induction of cell apoptosis (Inagaki et al., 2007; Schirmer et al., 2016), the promotion of plant growth (Elleuch et al., 2010), and the exertion of antiviral and antipathogenic properties (Zhu and Jin, 2015), underscoring its research potential and broad application prospects.

Nowadays, aromatic alcohols are primarily obtained either through chemical synthesis or extraction from plant materials. However, the chemical approach is environmentally unfriendly and often yields lower-grade products, whereas the extraction method suffers from high costs and unstable supply (Xu et al., 2017; Dubhashe et al., 2018; Yan et al., 2021; Wang et al., 2025). In response to these challenges, biosynthesis has attracted growing interest due to its eco-friendly nature and promising industrialization potential. *Saccharomyces cerevisiae*, a generally recognized as safe (GRAS) food-grade microorganism, is an ideal chassis cell for synthetic biology. Aromatic alcohols are biosynthesized in this strain via two metabolic pathways. The first is the Ehrlich pathway, which initiates from aromatic amino acids and proceeds through three enzymatic steps: (1) transamination to 2-oxoacids catalyzed by ARO8 and ARO9, (2) decarboxylation to aromatic aldehydes by ARO10, PDC1, PDC5 and PDC6, and (3) reduction to aromatic alcohols by ADH1–ADH5 and SFA1 (Dickinson et al., 2003; Gu et al., 2020; Liu et al., 2024). The second route is the *de novo* pathway, which converts glucose to aromatic alcohols through the following steps: (1) phosphoenolpyruvate (from glycolysis) and erythrose 4-phosphate (from pentose phosphate pathway) are condensed into 3-deoxy-D-arabino-heptulosonate-7-phosphate (DAHP) under the catalysis of ARO3 and ARO4; (2) DAHP is sequentially converted to chorismate through reactions catalyzed by the pentafunctional arom protein ARO1 and chorismate synthase ARO2; (3) for 2-PE biosynthesis, chorismate is converted to prephenate by ARO7, then to phenylpyruvate by PHA2, and finally enters the Ehrlich pathway for decarboxylation and reduction; for TOL biosynthesis, chorismate is transformed into tryptophan by enzymes TRP1–TRP5, before proceeding through the Ehrlich pathway (Hassing et al., 2019; Guo et al., 2020; Wu et al., 2024).

In *S. cerevisiae*, besides the core catalytic enzymes, the aromatic alcohol biosynthesis is also regulated by pathway-related regulatory proteins. For instance, transcriptional activator ARO80 and GATA factors (e.g., GLN3, GAT1) modulate aromatic alcohol biosynthesis by upregulating *ARO9* and *ARO10* gene expression (Cooper, 2002; Dai et al., 2021). The decarboxylation reaction in the Ehrlich pathway relies on thiamine pyrophosphate (TPP) as a coenzyme (Dai et al., 2021), meaning thiamine regulon genes (*THI*s) indirectly control aromatic alcohol production. This is supported by our previous work, where overexpression of the *THI4* gene significantly increased yields of 2-PE and TOL in *S. cerevisiae* (Gong et al., 2022). In addition, the reduction step in the Ehrlich pathway requires NADH. Lipoamide dehydrogenase (LPD1) catalyzes the oxidative regeneration of dihydrolipoamide coupled with NAD^+^ reduction to NADH. Thus, reduced 2-PE and TOL yields in the *LPD1* mutant may be linked to NADH availability (Dickinson et al., 2003).

The unfolded protein response (UPR) can be induced by endoplasmic reticulum (ER) stress (Read and Schröder, 2021; Hrach et al., 2023; Hong et al., 2024). ER stress arises from stimuli such as Ca^2+^ depletion, hypoxia, altered glycosylation, and viral infection, and ultimately results in the accumulation of unfolded or misfolded proteins in the ER lumen. In *S. cerevisiae*, the UPR is mediated exclusively through the IRE1 (Inositol-requiring enzyme 1) pathway (Read and Schröder, 2021). IRE1, an ER-localized type I transmembrane protein, functions as a key sensor for both ER stress and ER bilayer stress (Adams et al., 2019; Ho et al., 2020). Upon sensing either stress type, IRE1 is activated and acquires endonuclease activity, which enables it to specifically splice HAC1 mRNA and generate mature HAC1 protein (Cox et al., 2011). Mature HAC1 then binds to unfolded protein response elements in the promoters of UPR target genes, driving their expression to support processes including protein folding, degradation, and secretion (Halbleib et al., 2017; Read and Schröder, 2021). Within cells, a successful UPR restores normal protein folding homeostasis, while failure triggers cellular apoptosis (Read and Schröder, 2021). To date, no studies have reported an association between UPR and aromatic alcohol biosynthesis.

In this study, an unfolded protein response-inducible gene *ULI1* was disrupted and heterologously expressed in *S. cerevisiae*, revealing its involvement in the biosynthesis of 2-PE and TOL. Using transcriptomics and proteomics, we further elucidated the molecular mechanisms by which ULI1 regulates aromatic alcohol biosynthesis, aiming to provide theoretical support and genetic resources for efficient production of aromatic alcohols in *S. cerevisiae*. ULI1 overexpression may enhance aromatic alcohol biosynthesis in industrial fermentation. Nevertheless, critical factors governing strain stability and scaled production require ongoing investigation.

## 2 Materials and methods

### 2.1 Strains and culture conditions

The strains used in this study are listed in Supplementary Table S1. The wild-type *S. cerevisiae* strain KMLY1-2 (isolated from homemade sourdough; Gong et al., 2022) and its *MAT*a haploid derivative KMLY1-6 (obtained by ascospore selection) were cultured in YPD medium (1% yeast extract, 2% peptone, 2% dextrose). *S. cerevisiae* strain BY4741 was maintained in synthetic complete dropout medium without uracil (SC-URA; Coolaber, Beijing, China). For 2-PE and TOL production, all yeast strains were cultured in TM medium (30 g/L glucose, 5 g/L KH_2_PO_4_, 0.5 g/L MgSO_4_, 1 g/L NaCl) supplemented with 1.75 g/L L-phenylalanine (TM-1.75P) or 0.6 g/L L-tryptophan (TM-0.6T), respectively. *Escherichia coli* strain DH5α, used for plasmids construction, was grown in LB medium (1% tryptone, 0.5% yeast extract, 1% NaCl). Solid media were prepared by adding 2% agar to the corresponding broth. When required, antibiotics were supplemented as follows: 100 μg/mL ampicillin for *E. coli*, and 200 μg/mL geneticin plus 100 μg/mL nourseothricin for *S. cerevisiae*.

### 2.2 Gene sequence and phylogenetic analyses

Based on our previous RNA-seq data (Gong et al., 2022), two differentially expressed genes (DEGs; Supplementary Table S2), namely the unfolded protein response-inducible gene *ULI1* and the aldehyde dehydrogenase gene *ALD3*, were selected to investigate their effects on 2-PE and TOL production. The nucleotide sequences of *ULI1* and *ALD3* were obtained from the KMLY1-2 genome (Gong et al., 2022). Their deduced amino acid sequences, theoretical isoelectric points (pI), and molecular weights were predicted using EditSeq (DNASTAR, Madison, United States). Conserved functional domains in ULI1 and ALD3 proteins were identified using InterPro.^1^ SignalP 5.0, TargetP 2.0 and TMHMM 2.0^2^ were used to detect signal peptides, subcellular localization, and transmembrane helices in ULI1 and ALD3, respectively. Homologous proteins of ULI1 and ALD3 from different yeast species were downloaded from NCBI^3^ and aligned using Clustal X. A neighbor-joining tree was constructed using the MEGA 6.0 software package (Mega Limited, Auckland, New Zealand).

### 2.3 Genes disruption by CRISPR-Cas9 system

The oligonucleotides used in this study are listed in Supplementary Table S3. All gene disruption mutants were generated using the CRISPR-Cas9 gene editing system (Jessop-Fabre et al., 2016). To disrupt the *ULI1* gene, a single-guide RNA (sgRNA) targeting *ULI1* was designed using CHOPCHOP,^4^ and two complementary oligonucleotides (Sangon Biotech, Shanghai, China) containing a 20-bp spacer flanked by 15-bp homologous arms were annealed to form the dsDNA fragment. This fragment was cloned into the linearized pCfB3052 vector, amplified using primers 3052-F and 3052-R, via In-Fusion assembly with the ClonExpress MultiS One Step Cloning Kit (Vazyme Biotech, Nanjing, China), generating plasmid pCfB3052-uli1 (Supplementary Figure S1). The donor DNA for homologous recombination was constructed by overlap PCR using the *ULI1* upstream and downstream arms amplified from KMLY1-6 genomic DNA with primer pairs uli1-uF/uli1-uR and uli1-dF/uli1-dR. The Cas9-expressing plasmid pCfB2312 was co-transformed into KMLY1-6 with either pCfB3052-uli1 alone or pCfB3052-uli1 plus donor DNA. Positive transformants selected on geneticin and nourseothricin were verified by PCR and sequencing, ultimately obtaining the *ULI1* frameshift mutant Kmuli1 and knockout mutant KΔuli1. For *ALD3* disruption, two sgRNAs targeting *ALD3* were designed, and three cassettes containing two 20-bp spacers (Cassette I, amplified from pCfB3052-uli1 with F1/ald3-R1; Cassette II amplified from pCfB3052 with ald3-F2/ald3-R2; Cassette III, amplified from pCfB3052-uli1 with ald3-F3/R3) were In-Fusion cloned into the linearized pCfB3052 backbone, which was prepared using primers 3052c-F/3052c-R, generating plasmid pCfB3052-ald3 (Supplementary Figure S1). Donor DNA was constructed by combining the upstream and downstream arms of the *ALD3* gene, amplified from KMLY1-6 genomic DNA using primer sets ald3-uF/ald3-kuR and ald3-kdF/ald3-dR, respectively. Following co-transformation of KMLY1-6 with pCfB2312, pCfB3052-ald3, and the donor DNA, candidate colonies were isolated and verified by PCR, generating the *ALD3* knockout strain KΔald3.

### 2.4 Construction of *ULI1* chromosomal integration expression (CIE) strains

Jessop-Fabre et al. (2016) identified multiple genomic loci suitable for gene integration and expression in *S. cerevisiae* strain CEN.PK113-7D. The corresponding sites (X-3 and XII-3) in KMLY1-6, along with the *ALD3* gene, were selected as integration targets for *ULI1*. Plasmids pCfB3052-X-3 and pCfB3052-XII-3 (targeting X-3 and XII-3 loci, respectively) were constructed analogously to pCfB3052-ald3 but using different primers (F1/X-3-R1 and F1/XII-3-R1 for cassette I; X-3-F2/X-3-R2 and XII-3-F2/XII-3-R2 for cassette II; X-3-F3/R3 and XII-3-F3/R3 for cassette III) as listed in Supplementary Table S3. To assemble the *ULI1* expression cassette, the *ULI1* open reading frame (ORF) was PCR-amplified, digested with *Eco*RI and *Xho*I, and ligated into similarly digested pY26TEF-GPD, yielding pY26-uli1, from which the cassette was amplified using primers Uec-F and Uec-R. The upstream and downstream arms of X-3, XII-3, and *ALD3* were amplified using the primer pairs X-3-uF/X-3-uR and X-3-dF/X-3-dR, XII-3-uF/XII-3-uR and XII-3-dF/XII-3-dR, and ald3-uF/ald3-uR and ald3-dF/ald3-dR, respectively. Three donor DNAs were generated by overlap PCR, each fusing the *ULI1* cassette to flanking arms of either X-3, XII-3, or *ald3*. Finally, three *ULI1* CIE strains comprising KXuli1 (*uli1* integrated at X-3), KXIIuli1 (*ULI1* integrated at XII-3), and KΔald3uli1 (*uli1* replacing *ald3* ORF) were constructed following transformation, selection, and verification procedures equivalent to those used for KΔald3.

### 2.5 Construction of *ULI1* plasmid-based expression (PBE) strains

The plasmid pY26-uli1 was introduced into the yeast strain BY4741 by electroporation, and the *ULI1* PBE strain (BY4741/pY26-uli1) was selected on SC-URA plates. Strain BY4741 harboring the empty plasmid pY26TEF-GPD (BY4741/pY26) served as the control.

### 2.6 Determination and statistical analysis of 2-PE and TOL production

Frozen stock cultures of KMLY1-6 and its engineered derivatives were revived by incubation in 5 mL YPD medium at 30 °C and 150 rpm for 12 h, followed by inoculation (1% v/v) into 50 mL of fresh YPD medium for further cultivation under identical conditions. In parallel, strains BY4741/pY26 and BY4741/pY26-uli1 were pre-cultured and subsequently scaled up in SC-URA medium using the same growth parameters as KMLY1-6. All cells were harvested by centrifugation at 10,000 rpm for 5 min, washed twice with sterile ddH_2_O, and resuspended. The resulting suspension was inoculated into TM-1.75P and TM-0.6T media to an OD_600_ of 0.15, then cultivated for 48 h with 150 rpm shaking at 30 °C. Cell-free supernatant (CFS) was prepared by centrifugation (12,000 rpm, 10 min, 4 °C) followed by sterile filtration through a 0.22-μm filter (Millipore, Darmstadt, Germany), and was used to determine 2-PE and TOL content using high-performance liquid chromatography (HPLC; Agilent, Santa Clara, United States) according to Gong et al. (2022). To analyze the time course of 2-PE and TOL accumulation, the engineered strains Kmuli1 and BY4741/pY26-uli1, along with their controls, were cultured for 60 h. The concentrations of 2-PE and TOL in the CFSs were measured at 12-h intervals. Each experiment was conducted in at least triplicate and the data were expressed as mean ± standard deviation. Student’s *t*-test was performed using GraphPad Prism 10.1 (San Diego, United State), and significance was set at *p <* 0.05.

### 2.7 RNA isolation and RNA-seq analysis

Strains KMLY1-6, Kmuli1, BY4741/pY26 and BY4741/pY26-uli1 were cultured in TM-1.75P and TM-0.6T media for 48 h as described above, generating the following samples: K1.75P and K0.6T (for KMLY1-6), M1.75P and M0.6T (for Kmuli1), B1.75P and B0.6T (for BY4741/pY26), and U1.75P and U0.6T (for BY4741/pY26-uli1). Cells from these samples were harvested by centrifugation, and total RNA was immediately extracted using the Trizol reagent kit (Invitrogen, Carlsbad, United State). With three biological replicates per condition, a total of 24 high-quality RNAs were obtained, which were then used to construct RNA-seq libraries following the manufacturer’s protocol of the TruSeq RNA sample preparation kit (Illumina, San Diego, United State). The libraries were sequenced on an Illumina HiSeq 4000 system to generate 100 nt paired-end raw reads. Clean reads were obtained using a Perl program following the criteria described by Wu et al. (2020) and subsequently mapped to the KMLY1-2 genome with HISAT2 (Kim et al., 2015) under default parameters. Aligned reads were quantified using the fragment per kilobase of transcript per million mapped reads (FPKM) method (Pertea et al., 2016). DEGs between two groups were identified using DESeq2 (Love et al., 2014), with filtering thresholds set as | log_2_^(*fold* change)^| ≥ 1 and adjusted *p*-value < 0.05. The DEGs were classified and enriched by Gene Ontology (GO) and Kyoto Encyclopedia of Genes and Genomes (KEGG) pathway annotation. Heatmaps of gene and protein expression profiles were generated using R package.

### 2.8 Proteomic analysis

Total proteins from samples B1.75P, B0.6T, U1.75P, and U0.6T were extracted and trypsinized following the method described by He et al. (2020). Then, the peptides were desalted and analyzed using liquid chromatography-tandem mass spectrometry (LC-MS/MS; ThermoFisher Scientific, Waltham, United State) at Majorbio Bio-Pharm Technology Co., Ltd. (Shanghai, China). Briefly, the peptides were separated using a VanquishNeo instrument equipped with a uPAC High Throughput column (75 μm × 5.5 cm). A gradient elution was performed with mobile phases A (2% acetonitrile and 0.1% formic acid) and B (80% acetonitrile and 0.1% formic acid), using the program described in Supplementary Table S4. Data-independent acquisition (DIA) data were acquired using an Orbitrap Astral mass spectrometer operated in DIA mode. The detection was carried out over a mass range of 380–980 m/z (MS1), and 150–2000 m/z (MS2). The MS raw data were searched using Xcalibur 4.7 (ThermoFisher Scientific) against the UniProt-*Saccharomyces cerevisiae* database with the following parameters: protein false discovery rate (FDR) ≤ 0.01, peptide FDR ≤ 0.01, peptide confidence ≥ 99%, and extracted ion chromatogram (XIC) width ≤ 75 ppm. Differentially expressed proteins (DEPs) between groups were identified using the R package “*t*-test” with thresholds of fold change ≥ 1.2 or ≤ 0.83 and *p*-value < 0.05. These DEPs were further subjected to GO and KEGG enrichment analyses.

## 3 Results and discussion

### 3.1 Bioinformatic characterization of the *ULI1* and *ALD3* genes

The predicted ORFs of the *ULI1* and *ALD3* genes are 510 bp and 1521 bp in length, encoding polypeptides of 169 and 506 amino acid residues, respectively. The theoretical molecular weight and pI of ULI1 are 18.81 kDa and 6.35, while those of ALD3 are 55.42 kDa and 5.54 (Table 1). ULI1 contains a signal peptide and transmembrane (SP-TM) domain, with residues 1–19 comprising the SP sequence and residues 5–27 forming a TM helix (Figure 1A). This indicates that ULI1 is a membrane-bound protein, which is consistent with the previous finding that ULI1 may localize to the ER (Yofe et al., 2016). In contrast, ALD3 contains an ALDH-like domain (Figure 1A) and is predicted to be a cytosolic protein. Phylogenetic analysis showed that the homologous ULI1 and ALD3 from different yeasts were separated into two clades (Figure 1B). Notably, ULI1 homologs are found exclusively within the genus *Saccharomyces*, and exhibit significantly greater genetic divergence among themselves than do ALD3 homologs. This suggests that ULI1 is a genus-specific protein, and its evolutionary mechanism requires further investigation.

**TABLE 1:** Characteristics of ULI1 and ALD3 proteins.

Proteins	Molecular weight (kDa)	pI
ULI1	18.81	6.35
ALD3	55.42	5.54

**FIGURE 1:**
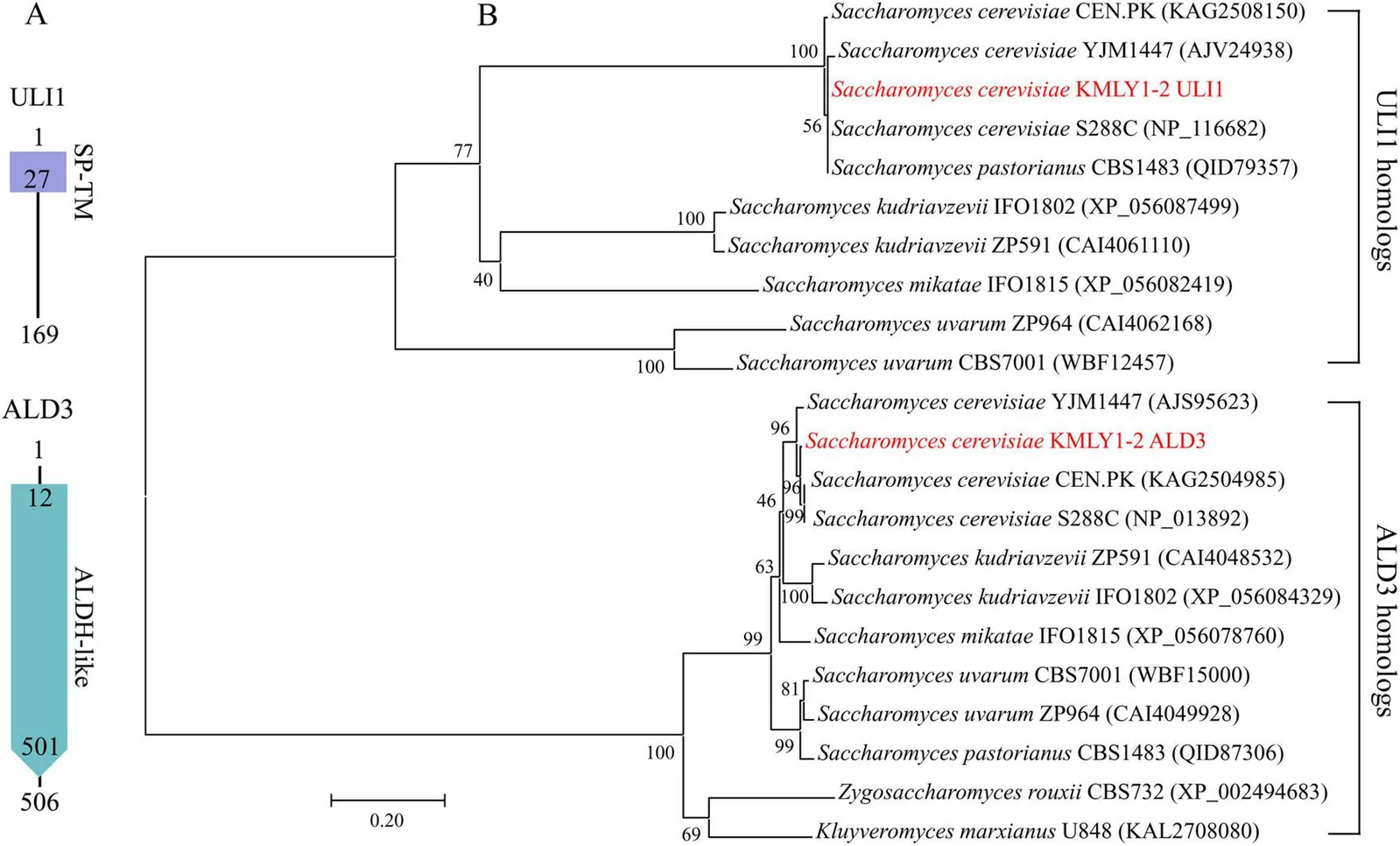
Domain organization and evolutionary analysis of ULI1 and ALD3 in yeasts. **(A)** Domain structure of ULI1 and ALD3 from KMLY1-2. Domain positions and counts are schematically shown (not to scale). **(B)** Neighbor-joining phylogenetic tree of ULI1 and ALD3 homologs. The numbers adjacent to the branches indicate the percentages at which given branches were supported in 1,000 bootstrap replications. GenBank accession numbers are displayed after the species name. KMLY1-2 ULI1 and ALD3 are highlighted in red.

### 3.2 Involvement of *ULI1* in 2-PE and TOL biosynthesis revealed by gene disruption assays

To test whether *ULI1* and *ALD3* are involved in aromatic alcohol biosynthesis, *ULI1* and *ALD3* of KMLY1-6 were disrupted by using CRISPR-Cas9 gene editing system. Sequence alignment analysis identified a single thymine (T) insertion in the *ULI1* gene between the 100th and 101st base pairs, which corresponds to the fifth nucleotide upstream of the PAM site (Supplementary Figure S2A). This insertion caused a frameshift mutation, resulting in a truncated 42-amino-acid peptide where 34 residues are identical to those encoded by the *ULI1* gene in the wild-type strain. This mutation stems from the repair of a Cas9-induced genomic double-strand break (DSB) via non-homologous end joining, an error-prone mechanism characterized by its propensity to introduce small indels (Chen et al., 2019), which directly resulted in the thymine insertion observed in the Kmuli1 strain. Additionally, *ULI1*- and *ALD3*- knockout mutants (KΔuli1 and KΔald3) were constructed using homology-directed recombination (HDR) with donor DNAs containing upstream and downstream homologous arms (Supplementary Figures S2B–D,G). One strain of each mutant was analyzed for aromatic alcohol production (Figures 2a,b). Disruption of *ULI1* significantly (*p* < 0.01) reduced 2-PE and TOL production. Kmuli1 and KΔuli1 produced 46.32 and 64.54% of the control (KMLY1-6) 2-PE levels, and 34.61 and 77.73% of its TOL levels, respectively. In contrast, *ALD3* knockout increased 2-PE and TOL production by 4.98 and 20.57%, respectively. These results indicate that *ULI1* and *ALD3* mediate the biosynthesis of 2-PE and TOL in *S. cerevisiae*. Current knowledge regarding ULI1 function is limited. Existing literature indicates that ULI1 localizes to the ER and is involved in and induced by the ER unfolded protein response (UPRER) (Metzger and Michaelis, 2009; Yofe et al., 2016). Given that TM-1.75P and TM-0.6T are nutrient-poor media, *S. cerevisiae* cultured in these media inevitably encounters starvation stress, which is likely to trigger the UPRER. Thus, ULI1 may be a UPRER component that influences the biosynthesis of 2-PE and TOL by aiding in maintaining cellular homeostasis and regulating cellular metabolism. The lower production of 2-PE and TOL in Kmuli1 compared to KΔuli1 (Figures 2a,b) may be related to the extent of *ULI1* disruption. Complete deletion in KΔuli1 might activate intracellular proteins functionally redundant to ULI1, conferring better cellular homeostasis than in Kmuli1, which retains a 34-amino-acid peptide. Regarding ALD3, it is proposed to function in a competing branch pathway by oxidizing the key aldehydic precursors (phenylacetaldehyde/indoleacetaldehyde) to their corresponding acids, thereby diverting flux away from the ADH-mediated production of 2-PE and TOL. This model explains the observed increase in these aromatic alcohols upon *ALD3* knockout. Since the *ULI1* frameshift exerts the most pronounced effect, Kmuli1 was selected for production dynamics analysis. As shown in Figures 2C,D, 2-PE and TOL levels increased gradually with time, but KMLY1-6 consistently had much higher yields than Kmuli1, with differences of 378.04–697.78 mg/L (2-PE) and 180.31–299.58 mg/L (TOL). These results further support *ULI1*’s involvement in 2-PE and TOL biosynthesis.

**FIGURE 2:**
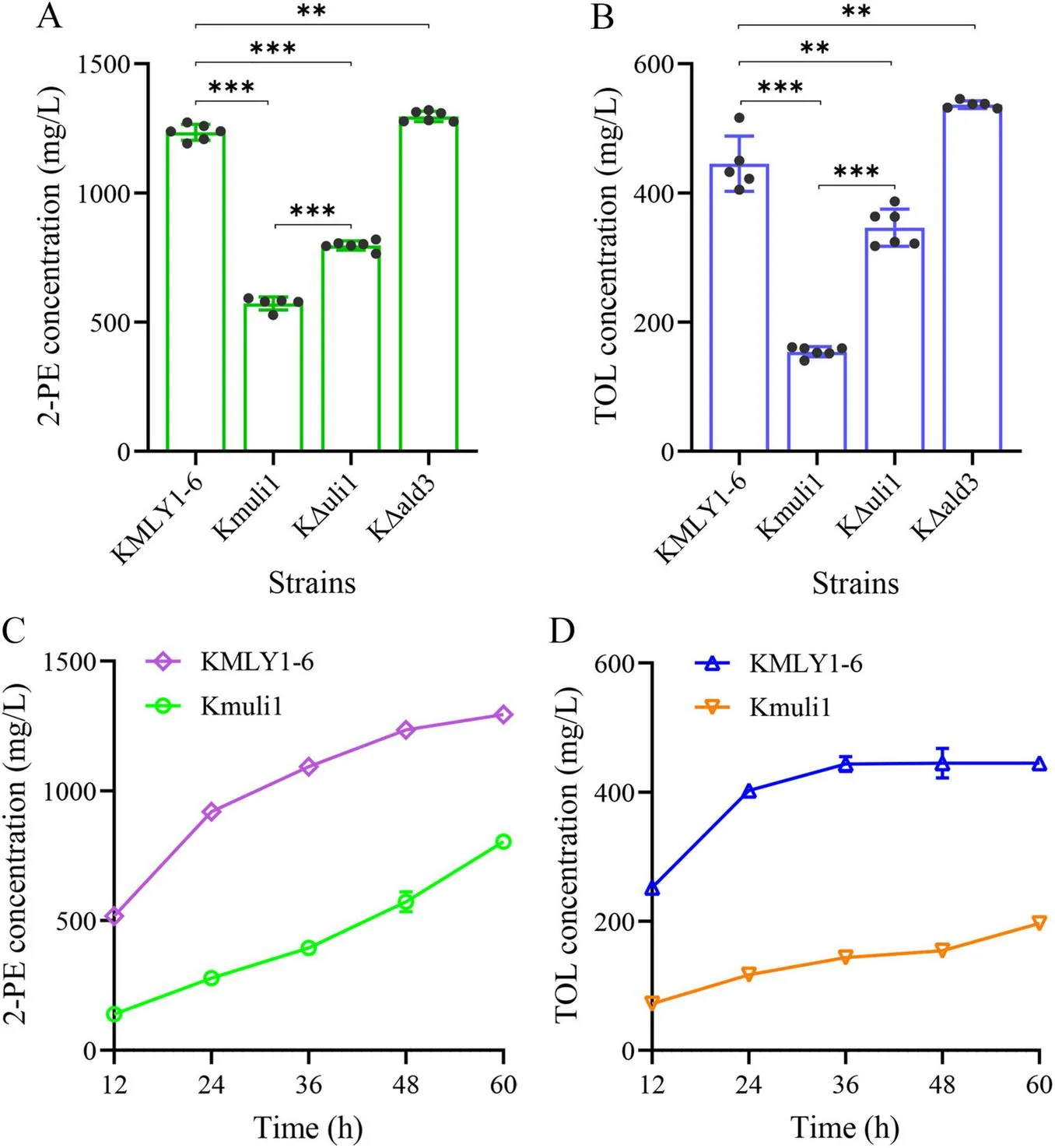
Effects of gene disruption on 2-phenylethanol (2-PE) and tryptophol (TOL) production. **(A)** 2-PE concentration of *ULI1* and *ALD3* disruption strains in TM-1.75P medium. **(B)** TOL concentration of *ULI1* and *ALD3* disruption strains in TM-0.6T medium. **(C)** 2-PE production kinetics curves of KMLY1-6 and *ULI1* mutant in TM-1.75P medium. **(D)** TOL production kinetics curves of KMLY1-6 and *ULI1* mutant in TM-0.6T medium. Error bars denote the standard deviation from three biological replicates. *p*-values were calculated using Student’s *t*-test (**p < 0.01, ***p < 0.001).

### 3.3 Involvement of *ULI1* in 2-PE and TOL biosynthesis confirmed by gene expression analysis

Given its stronger impact on 2-PE and TOL production than *ALD3* (Figures 2a,b), *ULI1* was selected for expression analysis. Using HDR, donor DNAs containing the *ULI1* cassette flanked by homologous arms were integrated into the X-3, XII-3 and *ALD3* loci of the KMLY1-6 chromosome. This resulted in size shifts of the corresponding PCR products from 2,782 to 3,262, 2,383 to 2,644, and 2,822 to 2,750 bp, respectively (Supplementary Figures S2D–G), confirming the successful construction of three *ULI1* CIE strains (KXuli1, KXIIuli1, KΔald3uli1). Yield analysis showed that *ULI1* overexpression at the X-3, XII-3 and *ALD3* loci increased 2-PE production by 12.91, 11.83, and 10.13%, and TOL production by 29.28, 28.44, and 25.76%, respectively (Figures 3a,b). These results indicate *ULI1* is involved in 2-PE and TOL biosynthesis in a *ULI1* abundance-dependent manner. The greater yield improvement of KΔald3uli1 compared to KMLY1-6 relative to KΔald3 is attributed to the combined effect of *ULI1* overexpression and *ALD3* knockout in KΔald3uli1. To verify whether *ULI1* plays the same role in the model *S. cerevisiae* strain, it was expressed in BY4741 using a plasmid-based system. As shown in Figures 3C,D, the yields of 2-PE and TOL increased with cultivation time. After 48 h of cultivation, the yields of 2-PE and TOL in BY4741/pY26-uli1 were significantly higher (*p* < 0.01) than those in the control strain BY4741/pY26, with respective increases of 38.80 and 30.86% observed at 60 h. These results further confirm that the involvement of *ULI1* in 2-PE and TOL biosynthesis in an abundance-dependent manner is applicable across different *S. cerevisiae* strains.

**FIGURE 3:**
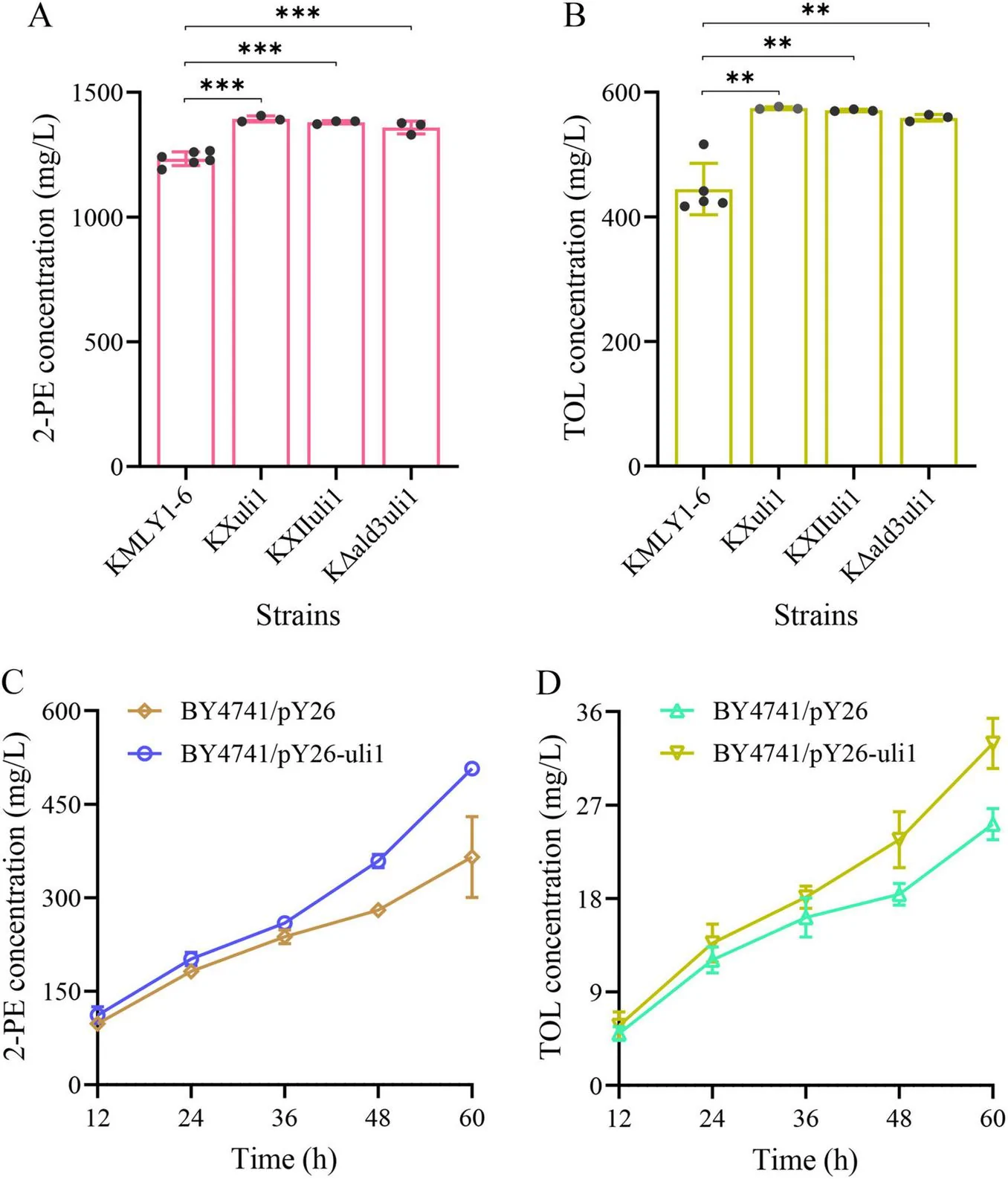
Effects of gene expression on 2-phenylethanol (2-PE) and tryptophol (TOL) production. **(A)** 2-PE concentration of *ULI1* chromosomal integration expression strains in TM-1.75P medium. **(B)** TOL concentration of *ULI1* chromosomal integration expression strains in TM-0.6T medium. **(C)** 2-PE production kinetics curves of BY4741/pY26 and BY4741/pY26-uli1 in TM-1.75P medium. **(D)** TOL production kinetics curves of BY4741/pY26 and BY4741/pY26-uli1 in TM-0.6T medium. Error bars denote the standard deviation from three biological replicates. *p*-values were calculated using Student’s *t*-test (^**^p < 0.01, ^***^p < 0.001).

### 3.4 Transcriptome sequencing and DEGs analysis of KMLY1-6 *ULI1* disruption

Given that the *ULI1* frameshift mutation resulted in the most significant decrease in 2-PE and TOL production, Kmuli1 and the wild-type strain KMLY1-6 were selected for comparative transcriptomics analysis. Principal component analysis (PCA) revealed that all samples clearly formed four distinct groups, with tight clustering of the three intra-group replicates, indicating high reproducibility (Figure 4A). Notably, groups K0.6T and M0.6T were proximal, while both were distant from K1.75P and M1.75P (which also separated from each other), suggesting that medium TM-1.75P had a more pronounced effect on the transcriptome than TM-0.6T. Following the criteria described in the methods, 3078 (486 upregulated, 2592 downregulated) and 212 (97 upregulated, 115 downregulated) DEGs were identified in the comparisons M1.75P *vs.* K1.75P and M0.6T *vs.* K0.6T, respectively (Figures 4B,C). Among these, 112 DEGs were shared between the two comparisons (Figure 4C), of which 75 displayed consistent up- or down-regulation patterns in both comparisons (Supplementary Table S5). These shared and consistently regulated DEGs may be closely associated with the observed reduction in both 2-PE and TOL production due to the *ULI1* frameshift mutation, and were selected for GO and KEGG enrichment analyses. As shown in Figure 4D, eight of the top 10 enriched GO terms were categorized as biological processes, among which the thiamine-containing compound metabolic process was the most enriched. Correspondingly, thiamine metabolism was identified as the most significantly enriched KEGG pathway (Figure 4E and Supplementary Table S5). In our previous study, we proved that thiamine regulon genes (e.g., *THI4*) were involved in TOL production (Gong et al., 2022). These findings collectively indicate that ULI1 likely regulates 2-PE and TOL biosynthesis by modulating thiamine metabolism.

**FIGURE 4:**
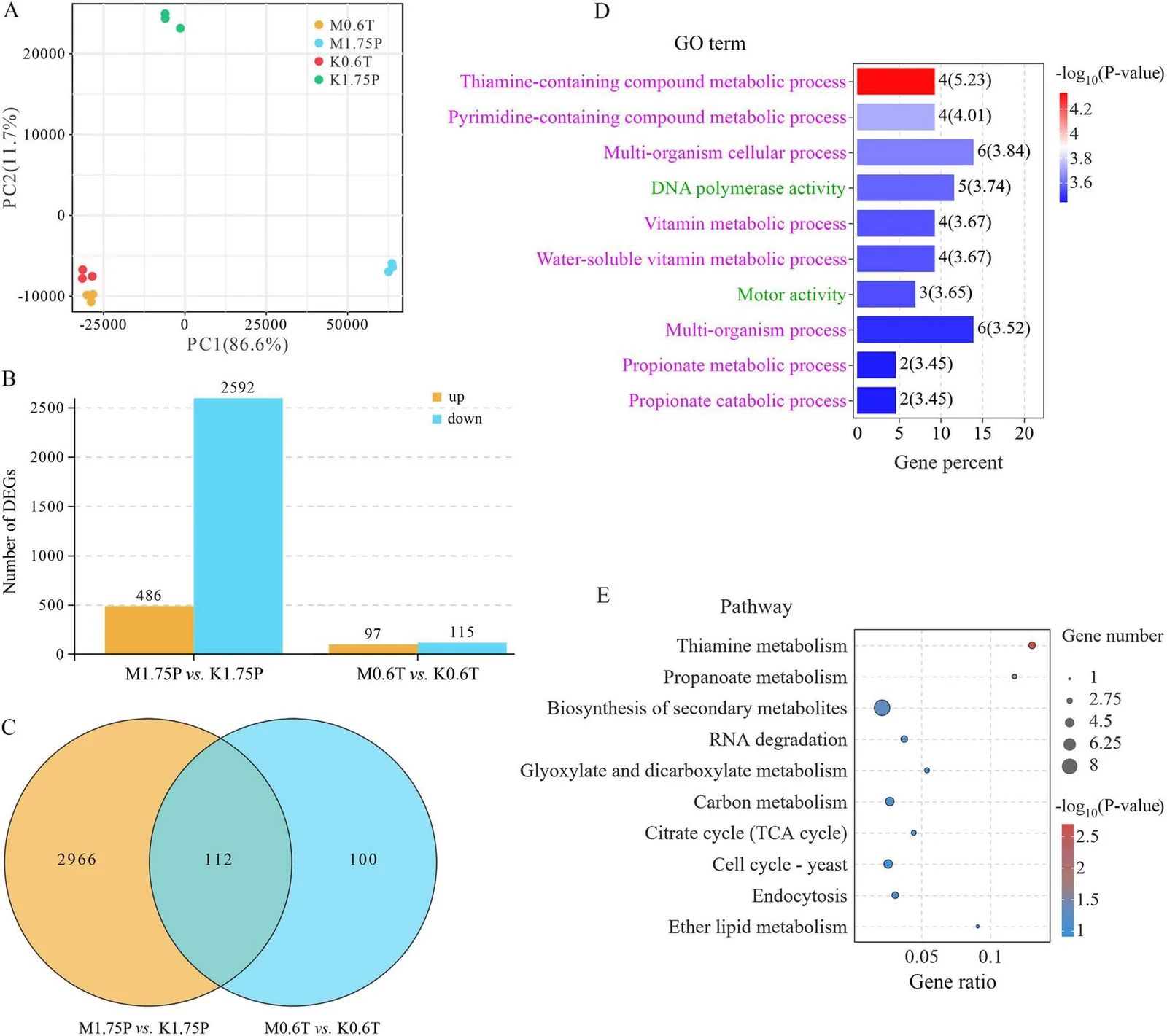
Statistics and enrichment analysis of differentially expressed genes (DEGs) between Kmuli1 and KMLY1-6 from transcriptome data. **(A)** Principal component analysis (PCA) derived score plot. **(B)** Number of DEGs. **(C)** Venn diagram of DEGs. **(D)** Top 10 Gene Ontology (GO) terms of shared DEGs with consistent up- or down-regulation in M1.75P *vs.* K1.75P and M0.6T *vs.* K0.6T. Pink terms denote biological processes and green those represent molecular functions. **(E)** Top 10 Kyoto Encyclopedia of Genes and Genomes (KEGG) enrichment pathways of shared DEGs with consistent up- or down-regulation in M1.75P *vs.* K1.75P and M0.6T *vs.* K0.6T. Three biological replicates were set for every sample (*n* = 3).

### 3.5 Transcriptome sequencing and DEGs analysis of BY4741 *ULI1* expression

To further characterize the function of the *ULI1* gene in yeast, comparative transcriptomic analysis was performed using strains BY4741/pY26 and BY4741/pY26-uli1. PCA revealed four clearly separated clusters, with tight grouping among three biological replicates, demonstrating high reproducibility (Figure 5A). The distinct separation between groups suggested that both culture media (TM-0.6T and TM-1.75P) and *ULI1* over-expression significantly influenced the transcriptomic profile. According to the thresholds described in the methods, 917 DEGs (167 upregulated, 750 downregulated) and 1002 DEGs (88 upregulated, 914 downregulated) were identified in the U1.75P *vs.* B1.75P and U0.6T *vs.* B0.6T comparisons, respectively (Figure 5B). Among these DEGs, 540 were shared between the two comparisons (Figure 5C), and all exhibited consistent expression trends (Supplementary Table S6). These common and stably regulated DEGs were likely closely related to the increased 2-PE and TOL production caused by the *ULI1* over-expression, and were therefore used for subsequent GO and KEGG enrichment analyses. As shown in Figure 5D, among the top 10 enriched GO terms, mitochondrion organization was the most significantly enriched term. The remaining nine terms included cellular macromolecule catabolic process, proteolysis involved in cellular protein catabolic process, macromolecule catabolic process, cellular protein catabolic process, modification-dependent protein catabolic process, organelle organization (biological processes, BP), as well as basal transcription machinery binding, basal RNA polymerase II transcription machinery binding, and protein binding (molecular functions, MF). Collectively, seven of the top 10 GO terms belonged to the BP category, while the other three were assigned to the MF category. In the KEGG pathway enrichment analysis, the top 10 pathways were proteasome, mitophagy-yeast, endocytosis, peroxisome, SNARE interactions in vesicular transport, basal transcription factors, non-homologous end-joining, ABC transporters, other glycan degradation, and longevity regulating pathway-multiple species. Functional classification of these KEGG pathways showed that four were involved in genetic information processing and three in cellular processes, with the remaining pathways distributed across metabolism and environmental information processing (Figure 5E and Supplementary Table S6). The convergent enrichment of mitochondrion organization (top GO term) and proteasome/mitophagy (top KEGG pathways) strongly indicates that *ULI1* overexpression triggers a coordinated cellular response centered on organelle remodeling and protein homeostasis in BY4741. These data demonstrate that ULI1 functions as a regulator of a pleiotropic network encompassing mitochondrial structure, protein degradation, organelle crosstalk, and transcriptional regulation, which likely underlies its reported effects on metabolic engineering and stress tolerance in yeast.

**FIGURE 5:**
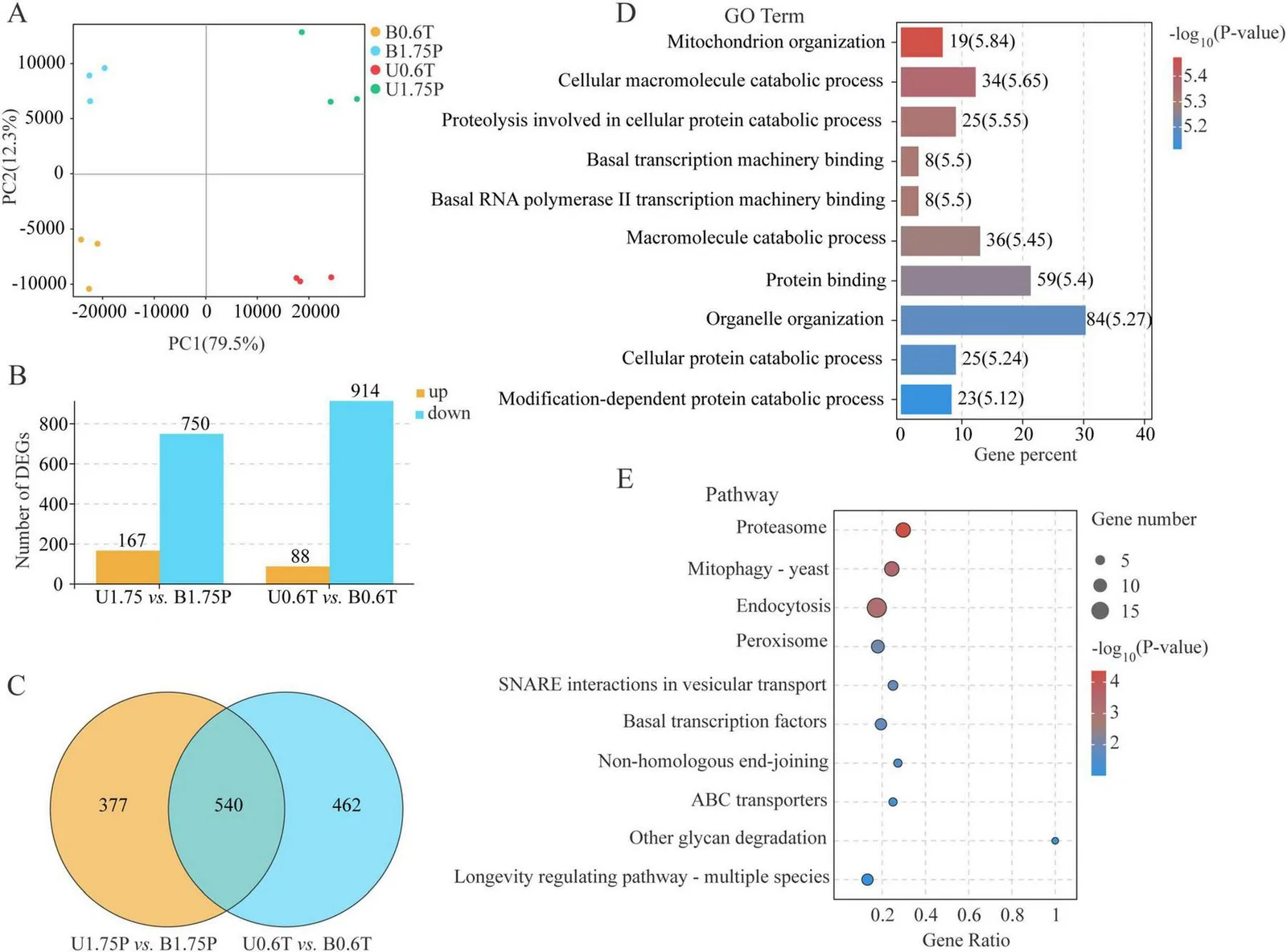
Statistics and enrichment analysis of differentially expressed genes (DEGs) between BY4741/pY26-uli1 and BY4741/pY26 from transcriptome data. **(A)** Principal component analysis (PCA) derived score plot. **(B)** Number of DEGs. **(C)** Venn diagram of DEGs. **(D)** Top 10 Gene Ontology (GO) terms of shared DEGs with consistent up- or down-regulation in U1.75P *vs.* B1.75P and U0.6T *vs.* B0.6T. **(E)** Top 10 Kyoto Encyclopedia of Genes and Genomes (KEGG) enrichment pathways of shared DEGs with consistent up- or down-regulation in U1.75P *vs.* B1.75P and U0.6T *vs.* B0.6T. Three biological replicates were set for every sample (*n* = 3).

### 3.6 Proteome sequencing and DEPs analysis of BY4741 *ULI1* expression

PCA showed that the sample replicates were closely clustered and *ULI1*-expressing samples (U1.75P and U0.6T) were clearly separated from their respective controls (Figure 6A), suggesting that the proteomic data were highly reproducible and *ULI1* expression significantly altered the cellular proteomic profile. A total of 4,472 proteins were identified, of which 2,258 and 2,345 were DEPs in the comparisons of U1.75P *vs.* B1.75P and U0.6T *vs.* B0.6T, respectively (Supplementary Table S7). In both comparisons, upregulated DEPs (1,353 and 1,313) outnumbered downregulated ones (905 and 1,032) significantly (Figures 6B,C). Given that ULI1 co-regulates the biosynthesis of both 2-PE and TOL (Figures 2, 3), we hypothesized that its expression would induce common proteomic changes. Therefore, 1,644 shared DEPs in the U1.75P *vs.* B1.75P and U0.6T *vs.* B0.6T comparisons were identified, of which 1,620 (984 up- and 636 down-regulated) exhibited a consistent expression trend across both comparisons (Figure 6D and Supplementary Table S7). These 1,620 DEPs were enriched into 522 GO terms (Supplementary Table S7). The top-enriched terms included biological process (e.g., peptide metabolic process, amide biosynthetic process), cellular component (e.g., mitochondrion, mitochondrial protein-containing complex), and molecular function (e.g., structural constituent of ribosome, oxidoreductase activity) (Figure 6E). The up- and down-regulated shared DEPs were subjected to KEGG enrichment analysis (Supplementary Table S7). As shown in Figures 6F,G, the up-regulated DEPs were significantly enriched in catabolic pathways including carbon metabolism, tryptophan metabolism, and citrate cycle; in contrast, the down-regulated DEPs were associated with anabolic pathways such as amino acid biosynthesis, other types of O-glycan biosynthesis, and monobactam biosynthesis. These findings suggest that *ULI1* expression enhances the utilization of exogenous nutrients while reducing non-essential biosynthesis to conserve energy. Since phenylalanine and tryptophan are key nutrients in TM-1.75P and TM-0.6T media, respectively, their extensive consumption will inevitably facilitate the production of metabolites like 2-PE and TOL.

**FIGURE 6:**
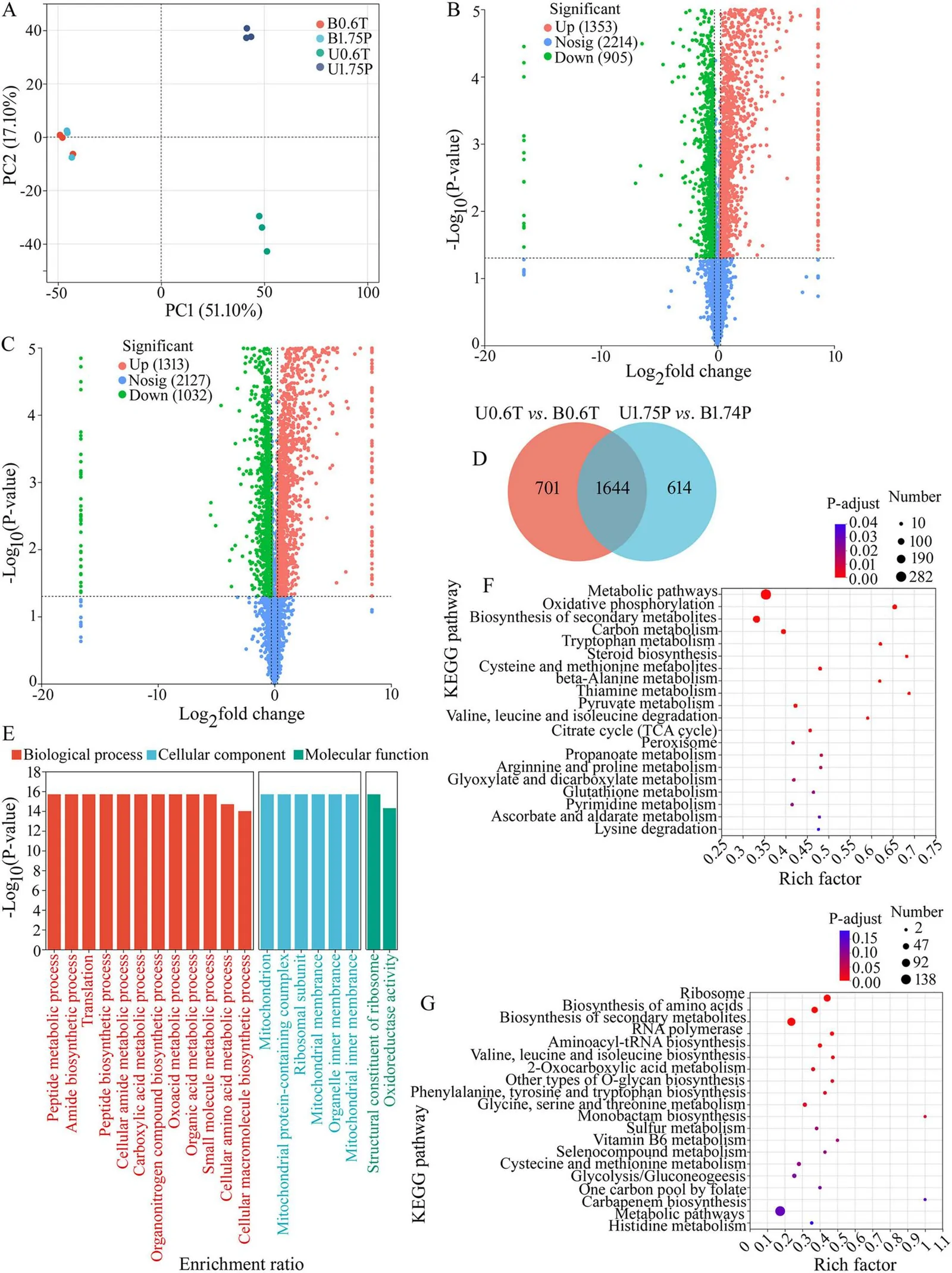
Statistics and enrichment analysis of differentially expressed proteins (DEPs) between BY4741/pY26-uli1 and BY4741/pY26 from proteome data. **(A)** Principal component analysis (PCA) derived score plot. **(B)** Volcano plot of DEPs from the comparison of U1.75P *vs.* B1.75P. **(C)** Volcano plot of DEPs from the comparison of U0.6T *vs.* B0.6T. **(D)** Venn diagram of DEPs from both comparisons. **(E)** Top 20 Gene Ontology (GO) terms of shared DEPs with consistent up- or down-regulation in both comparisons. **(F)** Top 20 Kyoto Encyclopedia of Genes and Genomes (KEGG) enrichment pathways of up-regulated shared DEPs in both comparisons. **(G)** Top 20 KEGG enrichment pathways of down-regulated shared DEPs in both comparisons. Three biological replicates were set for every sample (*n* = 3).

### 3.7 Heatmap cluster analysis

The genes and proteins associated with 2-PE and TOL *de novo* biosynthesis, regulation of Ehrlich pathway, and the unfolded protein response were subjected to heatmap cluster analysis. The transcriptomes of KMLY1-6 *ULI1* disruption and BY4741 *ULI1* overexpression exhibited no significant differential pattern between control samples (K0.6T, K1.75P, B0.6T, B1.75P) and experimental samples (M0.6T, M1.75P, U0.6T, U1.75P), respectively (Figures 7a,b). In contrast, the proteome of BY4741 *ULI1* overexpression showed an evident differential pattern between the corresponding control samples (B0.6T, B1.75P) and experimental samples (U0.6T, U1.75P) (Figure 7C). The results indicate that ULI1 mainly functions at the post-transcriptional translation stage or the post-translational protein folding stage, which supports that ULI1 was involved in the UPR (Metzger and Michaelis, 2009). Benisch et al. (2023) found that UPR regulation could optimize protein production. In our study, compared to the control samples B0.6T and B1.75P, many aromatic alcohols *de novo* synthesis enzymes (ARO2, ARO7, ARO9, ARO10, PDC5, ADH2, ADH4, and SFA1), plenty of thiamine regulon proteins (THI3, THI4, THI6, THI13, THI20, THI21, THI72, THI73, and THI80), ARO80 (the key positive regulation factor of ARO9 and ARO10), as well as LPD1 were up-regulated in the experimental samples U0.6T and U1.75P (Figure 7C). In experimental samples, numerous proteins with large changes compared to the control samples, such as ARO9 (8.32- to 16.84-fold), ARO10 (18.29- to 40.32-fold), PDC5 (21.71- to 41.90-fold), ADH2 (5.84- to 7.65-fold), ADH4 (69.44- to 196.82-fold), THI4 (31.83- to 77.91-fold), THI6 (8.80- to 14.12-fold), THI13 (47.54- to 118.40-fold), THI20 (14.10- to 18.89-fold), THI21 (14.19- to 18.43-fold), may contribute substantially to the improved biosynthesis of 2-PE and TOL. Transamination serves as the rate-limiting reaction of the Ehrlich pathway (Iraqui et al., 1999; Dai et al., 2021). The upregulation of ARO9 in the ULI1 overexpression groups U0.6T and U1.75P may enhance the Ehrlich pathway for aromatic alcohol biosynthesis. Meanwhile, the increased abundances of decarboxylase ARO10 and PDC5, as well as alcohol dehydrogenase ADH2, ADH4 and SFA1, further facilitate the progression of the Ehrlich pathway. Decarboxylation reaction in this pathway is strictly TPP-dependent and rate-limiting for 2-PE and TOL biosynthesis, and TPP acts as an essential coenzyme to stabilize catalytic intermediates and sustain decarboxylase activity (Romagnoli et al., 2012; Dai et al., 2021; Gong et al., 2022). Accordingly, the upregulation of thiamine regulatory proteins in experimental groups also indirectly promotes the synthesis of aromatic alcohols. The transcription factor ARO80 can activate the transcription of *ARO9* and *ARO10* during aromatic alcohol biosynthesis (Iraqui et al., 1999). Therefore, the upregulated expression of ARO80 in experimental samples may promote the transcription of *ARO9* and *ARO10*, thereby indirectly enhancing the Ehrlich pathway. The LPD1, which is involved in NADH generation, was also elevated in *ULI1*-overexpressing strains. Given that the reduction reaction of the Ehrlich pathway is NADH-dependent (Dickinson et al., 2003), the upregulation of LPD1 could further promote this metabolic pathway. In conclusion, ULI1 promotes the biosynthesis of aromatic alcohols (2-PE and TOL) by reinforcing the Ehrlich pathway.

**FIGURE 7:**
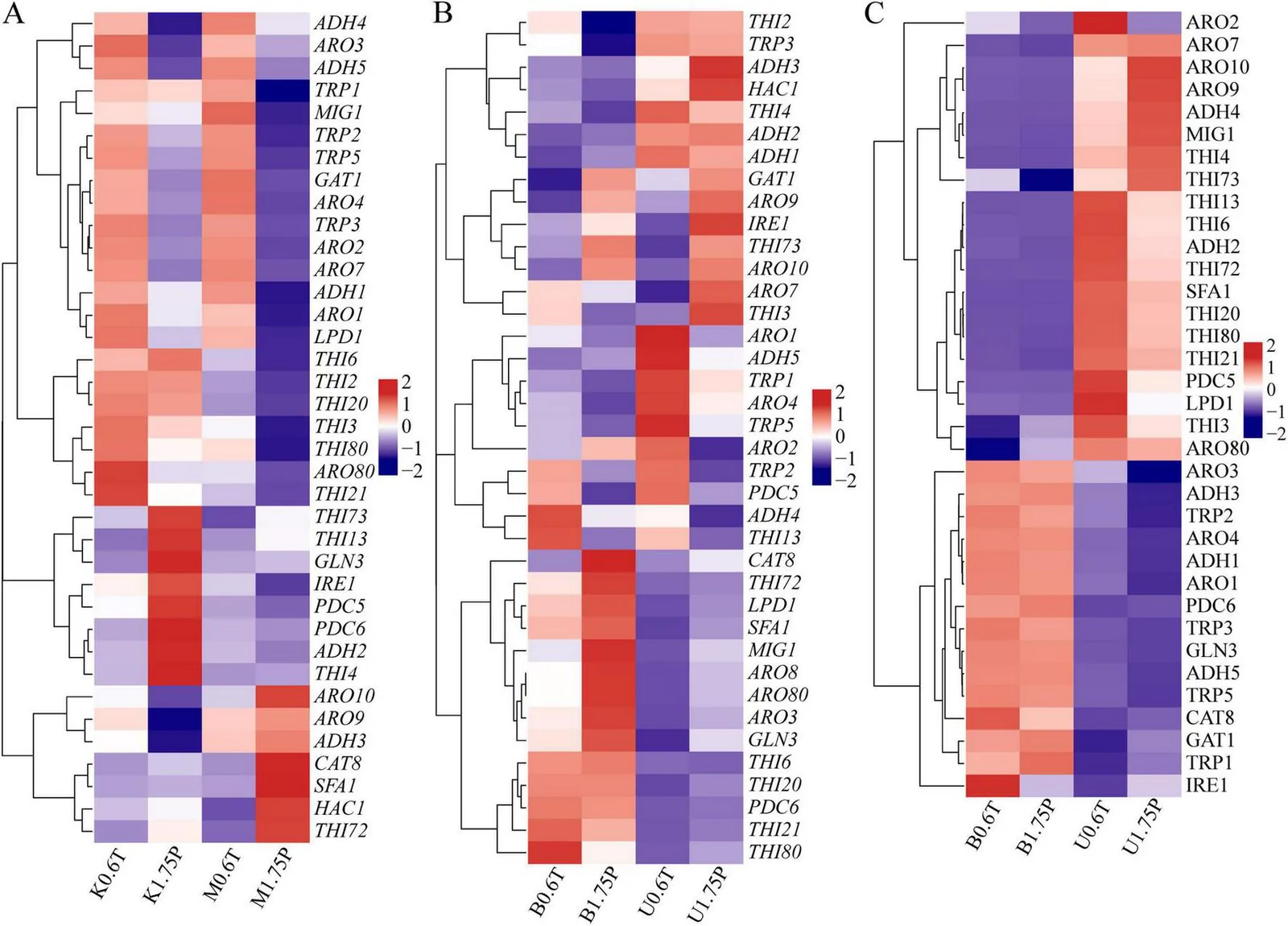
Heatmap profiling of genes involved in *de novo* biosynthesis of 2-phenylethanol (2-PE) and tryptophol (TOL), Ehrlich pathway regulation, and the unfolded protein response in *uli1*-disrupted KMLY1-6 **(A)** and *ULI1*-overexpressing BY4741 **(B)**. Heatmap profiling of proteins associated with the aforementioned biosynthetic and regulatory pathways in the *ULI1*-overexpressing BY4741 **(C)**. Three biological replicates were set for every sample (*n* = 3).

### 3.8 Omics insight into the regulatory role of ULI1

Transcriptomic analysis of the *ULI1* frameshift mutant showed that DEGs were highly enriched in thiamine metabolism, suggesting that ULI1 modulates 2-PE and TOL biosynthesis by regulating thiamine-related pathways. In *ULI1*-overexpressing cells, transcriptomic profiling revealed prominent enrichment in mitochondrial organization, proteasome function, mitophagy, and vesicular transport, indicating that ULI1 may maintain organelle structure and protein homeostasis. Proteomic analysis further showed that ULI1 overexpression tended to elevate the abundance of proteins associated with carbon metabolism, tryptophan metabolism, and the TCA cycle, whereas an overall decline was observed for proteins participating in anabolism including amino acid biosynthesis. This metabolic rearrangement may promote efficient utilization of exogenous nutrients, reduces energy consumption on unnecessary biosynthesis, and redirects metabolic flux toward aromatic alcohol production. Under nutritional starvation and ER stress, we propose that the ER-localized ULI1 orchestrates a global regulatory network by modulating thiamine metabolism, preserving mitochondrial architecture and cellular protein homeostasis, promoting catabolic pathways while restraining anabolic processes, and coordinating stress responses with metabolic flux redistribution, thereby enhancing the biosynthesis of 2-PE and TOL. This study identified the regulatory role of ULI1 in aromatic alcohol biosynthesis, while its molecular interactions remain unclear. ULI1 may act as a regulatory mediator to modulate yeast metabolism. Future studies will be carried out to identify its direct targets, further elucidating its regulatory mechanism for targeted metabolic engineering.

## 4 Conclusion

In this study, we characterized the bioinformatic features and regulatory function of ULI1, a genus-specific ER membrane protein in *Saccharomyces*. Gene knockout and overexpression experiments demonstrated that ULI1 positively regulates the biosynthesis of 2-PE and TOL in a dosage-dependent manner, whereas ALD3 negatively affects aromatic alcohol production by competing for key aldehyde precursors. Multi-omics analyses revealed that ULI1 does not directly catalyze aromatic alcohol synthesis but may function as a pleiotropic regulator connecting ER stress responses, organelle homeostasis, and central metabolism. ULI1 may coordinate thiamine and mitochondrial metabolism, enhance catabolic flux, and repress non-essential anabolic processes, thereby optimizing energy utilization and redirecting metabolic precursors toward 2-PE and TOL production. Our results suggest that ULI1 may serve as a novel regulatory hub linking stress tolerance, cellular homeostasis, and aromatic alcohol biosynthesis in yeast. The proposed regulatory role of *ULI1* in 2-PE and TOL biosynthesis remains to be fully verified. This work provides mechanistic insights and a promising target for future metabolic engineering strategies aimed at improving the industrial production of high-value aromatic alcohols and other valuable alcohols in *S. cerevisiae*.

## Data Availability

The datasets presented in this study can be found in online repositories. The names of the repository/repositories and accession number(s) can be found in the article/Supplementary material. Sequence data of this study have been deposited in the Short Read Archive under accession numbers SRR38241256–SRR38241279.

## Footnotes

1
http://www.ebi.ac.uk/interpro/search/sequence/

2
http://www.cbs.dtu.dk/services/

3
https://www.ncbi.nlm.nih.gov/

4
https://chopchop.cbu.uib.no

## Supplementary material

Supplementary Figure S1 Schematic diagram of gene-editing plasmids assembly.

Supplementary Figure S2 Verification of gene engineering strains. **(A)** Sequence alignment of the *ULI1* gene in KMLY1-6 and Kmuli1. **(B)** Schematic diagram of *ULI1* gene knockout and primers used for PCR verification. **(C)** Agarose gel electrophoresis of PCR products for verifying gene deletion events. M: DNA marker; Lanes 1–4: KΔuli1 strains; Lane 5: KMLY1-6. The PCR products from KΔuli1s and KMLY1-6 were 1,436 and 1,946 bp in length, respectively. **(D)** Schematic diagram of *ALD3* gene knockout, *ULI1* expression cassette integration, and primers used for PCR verification. **(E)** Schematic diagram of *ULI1* expression cassette integration at the X-3 site and primers used for PCR verification. **(F)** Schematic diagram of *ULI1* expression cassette integration at the XII-3 site and primers used for PCR verification. **(G)** Agarose gel electrophoresis of PCR products for verifying genomic integration events, amplified using the primers shown in **(D-F)**. M: DNA marker; Lanes 1: KΔald3 (1,301 bp); Lane 2: KΔald3uli1 (2,750 bp); Lane 4: KΔuli1 (3,262 bp); Lane 3, 5, and 6: KMLY1-6 (2,822 bp, 2,782 bp, 2,383 bp); Lane 7: KXIIuli1 (2,644 bp).
