# Supplementary material for: An unfolded protein response-inducible protein (ULI1) mediates aromatic alcohols biosynthesis through reinforcing the Ehrlich pathway in Saccharomyces cerevisiae
Source: Front Microbiol. 2026 Sep 14;17:1934502. doi: 10.3389/fmicb.2026.1934502 (PMC13617065; doi:10.3389/fmicb.2026.1934502)
Supplement: Supplementary file 1 [file Data_Sheet_1.zip › Supplementary Tables 1-4.docx]

**Supplementary TABLE S1** Strains and plasmids used in this study.

| Strain or plasmid | Genotype or description | Source |
| --- | --- | --- |
| Strains | | |
| *S. cerevisiae* KMLY1-2 | Wild-type strain, isolated from homemade sourdough | Heze, China |
| *S. cerevisiae* KMLY1-6 | KMLY1-2 *MAT*a haploid derivative | This study |
| *S. cerevisiae* Kmuli1 | KMLY1-6 *ULI1* gene frameshift mutant, generated by CRISPR-Cas9 gene editing | This study |
| *S. cerevisiae* KΔuli1 | KMLY1-6 *ULI1* gene knockout mutant, generated by CRISPR-Cas9 gene editing | This study |
| *S. cerevisiae* KΔald3 | KMLY1-6 *ALD3* gene knockout mutant, generated by CRISPR-Cas9 gene editing | This study |
| *S. cerevisiae* KⅩuli1 | KMLY1-6 X-3::*ULI1*, generated by insertion of the *ULI1* cassette at site X-3 | This study |
| *S. cerevisiae* KXIIuli1 | KMLY1-6 XII-3::*ULI1*, generated by insertion of the *ULI1* cassette at site XII-3 | This study |
| *S. cerevisiae* KΔald3uli1 | KMLY1-6 Δ*ald3*::*ULI1*, generated by replacing the *ALD3* ORF with the *ULI1* cassette | This study |
| *S. cerevisiae* BY4741 | *MAT*a *his3*Δ1 *leu2*Δ0 *met15*Δ0 *ura3*Δ0 | BNCC |
| *S. cerevisiae* BY4741/pY26-uli1 | BY4741 containing plasmid pY26-uli1 | This study |
| *S. cerevisiae* BY4741/pY26 | BY4741 containing empty plasmid pY26TEF-GPD | This study |
| *E. coli* DH5α | F^-^ endA1 glnV44 thi-1 recA1 relA1 gyrA96 deoR nupG φ80dlacZΔM15 Δ(lacZYA-argF)U169 hsdR17(rK^-^, mK^+^) λ^-^ | TaKaRa |
| Plasmids | | |
| pCfB2312 | *S. cerevisiae* episomal plasmid for Cas9 expression, Amp^R^, Neo^R^ | Addgene |
| pCfB3052 | *S. cerevisiae* episomal plasmid for sgRNAs expression, Amp^R^, Nat^R^ | Addgene |
| pCfB3052-uli1 | pCfB3052 containing one sgRNA targeting the *ULI1* gene, Amp^R^, Nat^R^ | This study |
| pCfB3052-ald3 | pCfB3052 containing two sgRNAs targeting the *ALD3* gene, Amp^R^, Nat^R^ | This study |
| pCfB3052-Ⅹ-3 | pCfB3052 containing two sgRNAs targeting the X-3 site, Amp^R^, Nat^R^ | This study |
| pCfB3052-ⅩII-3 | pCfB3052 containing two sgRNAs targeting the XII-3 site, Amp^R^, Nat^R^ | This study |
| pY26TEF-GPD | *S. cerevisiae* gene expression plasmid, 2 μm, *ura3*, P_GPD_/T_CYC1_, P_TEF1_/T_ADH1_, Amp^R^ | BioVector |
| pY26-uli1 | pY26TEF-GPD containing the *ULI1* coding sequence between P_GPD_ and T_CYC1_ | This study |

**Supplementary TABLE S2** Expression levels of *ULI1* and *ALD3* genes from our previous transcriptomic analysis.

| Gene | TM FPKM | TM-1.75P FPKM | TM-0.6T FPKM | TM-PT ^a^ FPKM | log_2_(TM-1.75P/TM) | FDR | log_2_(TM-0.6T/TM) | FDR | log_2_(TM-PT/TM) | FDR |
| --- | --- | --- | --- | --- | --- | --- | --- | --- | --- | --- |
| *ULI1* | 1.29 | 81.10 | 397.29 | 96.84 | 5.97 | 1.43E-115 | 8.27 | 4.85E-186 | 6.23 | 1.47E-28 |
| *ALD3* | 61.95 | 549.39 | 80.33 | 296.73 | 3.15 | 1.84E-106 | 0.37 | 1.86E-3 | 2.26 | 5.81E-36 |

^a^ TM-PT: TM medium supplemented with 1.75 g/L L-phenylalanine and 0.6 g/L L-tryptophan.

**Supplementary TABLE S3** Oligonucleotides used in this study.

| Oligos | Sequence (5'-3') ^a^ | Description |
| --- | --- | --- |
| Oligo-1 | GAAAGATAAATGATCTTTCCGCCATCAAATCTCAGGTTTTAGAGCTAGAA | Annealing to form a double-stranded DNA fragment |
| Oligo-2 | TTCTAGCTCTAAAACCTGAGATTTGATGGCGGAAAGATCATTTATCTTTC |  |
| 3052-F | GTTTTAGAGCTAGAAATAGCAAGTT | Amplifying pCfB3052 to obtain the linearized plasmid |
| 3052-R | GATCATTTATCTTTCACTGCGGAGA |  |
| uli1-uF | GAACGATAACGGTTCTTCTCCCAAC | Amplifying the 5' flanking fragment of the *ULI1* gene |
| uli1-uR | CACCTTTTCGAAATTTGACACTGCT |  |
| uli1-dF | AATTTCGAAAAGGTGTTGACATGTCATTTTTTGG | Amplifying the 3' flanking fragment of the *ULI1* gene |
| uli1-dR | AGTAAATCAAATTGATAAAAAAAAG |  |
| uli1-sF | TTTTTTCTGATCGTTGTATAT | Using for sequencing the *ULI1* gene frameshift mutant |
| uli1-sR | TTGTCTCTTGTGGTGTTGTAG |  |
| uli1-pF | AGGATATGGGAACCCACAGAAGC | Verifying the *ULI1* gene knockout mutant |
| uli1-pR | AAACCTTGATTGCCCTTGACG |  |
| F1 | GCGATAGGGAACAAAAGCTGGAGCT | Amplifying pCfB3052-uli1 to obtain cassette Ⅰ targeting *ald3*, X-3 and XII-3, respectively |
| ald3-R1 | TTGTGGGGAAAGCTTTAGGAGATCATTTATCTTTCACTGCGGAGA |  |
| Ⅹ-3-R1 | CATGGAAGGTCGATCTCTTCGATCATTTATCTTTCACTGC |  |
| Ⅻ-3-R1 | CGGCTGATTCCTATCAATATGATCATTTATCTTTCACTGC |  |
| ald3-F2 | TCCTAAAGCTTTCCCCACAAGTTTTAGAGCTAGAAATAGCAAGTT | Amplifying pCfB3052 to obtain cassette ⅠI targeting *ALD3* |
| ald3-R2 | TGTGGATCTTTCATTGGATAGATCATTTATCTTTCACTGCGGAGA |  |
| ald3-F3 | TATCCAATGAAAGATCCACAGTTTTAGAGCTAGAAATAGCAAGTT | Amplifying pCfB3052-uli1 to obtain cassette ⅠII targeting *ALD3*, X-3 and XII-3, respectively |
| Ⅹ-3-F3 | GAAAAGTGGCCTTAGAAACGGTTTTAGAGCTAGAAATAGC |  |
| Ⅻ-3-F3 | GTATGGTAATGCGAATGAGCGTTTTAGAGCTAGAAATAGC |  |
| R3 | GCGATTAACTAATTACATGACTCGA |  |
| 3052c-F | TCGAGTCATGTAATTAGTTAATCGC | Amplifying pCfB3052 to obtain the linearized plasmid |
| 3052c-R | AGCTCCAGCTTTTGTTCCCTATCGC |  |
| ald3-uF | TCTACAGTACCTTTCTTCCACGCCC | Amplifying the 5' flanking fragment of the *ALD3* gene |
| ald3-kuR | CTTCCTTTTGGCTAATTTTCTAAAT |  |
| ald3-uR | ATGGCGAGTATTGATAATGACTTCCTTTTGGCTAATTTTCTAAAT |  |
| ald3-kdF | GAAAATTAGCCAAAAGGAAGTTCGTTACGTAGTTATATAAATGTT | Amplifying the 3' flanking fragment of the *ALD3* gene |
| ald3-dF | GCTCGAAGGCTTTAATTTGCTTCGTTACGTAGTTATATAAATGTT |  |
| ald3-dR | AAGCCGTTATTAGTTTTGCTGATTT |  |
| ald3-pF | AATAGCTTAGCCTGACGACG | Verifying the *ALD3* gene knockout mutant |
| ald3-pR | TGATTCTTGTCTGCCCTTCTA |  |
| Ⅹ-3-F2 | GAAGAGATCGACCTTCCATGGTTTTAGAGCTAGAAATAGC | Amplifying pCfB3052 to obtain cassette ⅠI targeting X-3 |
| Ⅹ-3-R2 | CGTTTCTAAGGCCACTTTTCGATCATTTATCTTTCACTGC |  |
| Ⅻ-3-F2 | ATATTGATAGGAATCAGCCGGTTTTAGAGCTAGAAATAGC | Amplifying pCfB3052 to obtain cassette ⅠI targeting XII-3 |
| Ⅻ-3-R2 | GCTCATTCGCATTACCATACGATCATTTATCTTTCACTGC |  |
| uli1-eF | ATgaattcATGACGCCCTATGCAGTAG | Amplifying the coding sequence of the *ULI1* gene |
| uli1-eR | GTctcgagTCACTTTCCAGAGCTATAA |  |
| Uec-F | TCATTATCAATACTCGCCATTTCAA | Amplifying the *ULI1* expression cassette from pY26-uli1 |
| Uec-R | GCAAATTAAAGCCTTCGAGCGTCCC |  |
| X-3-uF | TTTTGGGTGATGCCTCTTTAGG | Amplifying the 5' flanking fragment of the X-3 site |
| X-3-uR | ATGGCGAGTATTGATAATGAGAGTATTGCCTGTGGACAACAGTAAC |  |
| X-3-dF | GCTCGAAGGCTTTAATTTGCGTAGGGTGAAAGTTCCTTTGTGCT | Amplifying the 3' flanking fragment of the X-3 site |
| X-3-dR | CGCAATATGACCTTGTCTGTGG |  |
| X-3-pF | ACCGTGGCATCCCAAGTAGAC | Verifying integration of the *ULI1* gene at X-3 site |
| X-3-pR | ATGGTTGAAAATGCCCCTGTC |  |
| XII-3-uF | ATTAGAAATGGAGCATACGAGCAAG | Amplifying the 5' flanking fragment of the XII-3 site |
| XII-3-uR | ATGGCGAGTATTGATAATGAGGAAGCGGAAATGCAGGAGT |  |
| XII-3-dF | GCTCGAAGGCTTTAATTTGCAAGTGCGACTGGAATTAGAAGAATG | Amplifying the 3' flanking fragment of the XII-3 site |
| XII-3-dR | CTTATGTGGGTATAGTAGCGGGTCT |  |
| XII-3-pF | CTTACCATGACTTAGCCGTTCGT | Verifying integration of the *ULI1* gene at XII-3 site |
| XII-3-pR | GGCACATTAGAAGCCCTTGATT |  |

^a^ The underlined nucleotides represent homologous arms; blue letters indicate the sgRNA1 spacer and red letters denote the sgRNA2 spacer; lowercase nucleotides mark restriction enzyme sites *Eco*RI and *Xho*I.

**Supplementary TABLE S4** Liquid chromatography elution gradient.

| Time (min) | A (%) | B (%) | Time (min) | A (%) | B (%) |
| --- | --- | --- | --- | --- | --- |
| 0 | 96 | 4 | 3.1 | 77.5 | 22.5 |
| 0.1 | 92 | 8 | 4.6 | 55 | 45 |
| 1 | 87.5 | 12.5 | 5 | 1 | 99 |
| 1.1 | 87.4 | 12.6 | 6.5 | 0 | 100 |
